# Post-Tooth Extraction Bacteraemia: A Randomized Clinical Trial on the Efficacy of Chlorhexidine Prophylaxis

**DOI:** 10.1371/journal.pone.0124249

**Published:** 2015-05-08

**Authors:** Mario Barbosa, Isabel Prada-López, Maximiliano Álvarez, Barbas Amaral, Casares-De-Cal María de los Angeles, Inmaculada Tomás

**Affiliations:** 1 School of Dentistry, Instituto Superior de Ciências da Saúde-Norte, Centro de Investigação de Ciências da Saúde, Gandra, Portugal; 2 Oral Sciences Research Group, Special Needs Unit, School of Medicine and Dentistry, University of Santiago de Compostela, Santiago de Compostela, Spain; 3 Department of Microbiology, CHUVI, Xeral-Cíes Hospital, IBIV, Vigo, Spain; 4 Department of Stomatology and Maxilo-Facial Surgery, St. António General Hospital, Oporto, Portugal; Cardiff University, UNITED KINGDOM

## Abstract

**Objectives:**

To investigate the development of post-extraction bacteraemia (PEB) after the prophylactic use of chlorhexidine (CHX).

**Patients and Methods:**

A total of 201 patients who underwent a tooth extraction were randomly distributed into four groups: 52 received no prophylaxis (CONTROL), 50 did a mouthwash with 0.2% CHX before the tooth extraction (CHX-MW), 51 did a mouthwash with 0.2% CHX and a subgingival irrigation with 1% CHX (CHX-MW/SUB_IR) and 48 did a mouthwash with 0.2% CHX and a continuous supragingival irrigation with 1% CHX (CHX-MW/SUPRA_IR). Peripheral venous blood samples were collected at baseline, 30 seconds after performing the mouthwash and the subgingival or supragingival irrigation, and at 30 seconds and 15 minutes after completion of the tooth extraction. Blood samples were analysed applying conventional microbiological cultures under aerobic and anaerobic conditions performing bacterial identification of the isolates.

**Results:**

The prevalences of PEB in the CONTROL, CHX-MW, CHX-MW/SUB_IR and CHX-MWSUPRA_IR groups were 52%, 50%, 55% and 50%, respectively, at 30 seconds and 23%, 4%, 10% and 27%, respectively, at 15 minutes. The prevalence of PEB at 15 minutes was significantly higher in the CONTROL group than in the CHX-MW group (23% *versus* 4%; *p* = 0.005). At the same time, no differences were found between CONTROL group and CHX-MW/SUB_IR or CHX-MW/SUPRA_IR groups. Streptococci (mostly viridans group streptococci) were the most frequently identified bacteria (69–79%).

**Conclusions:**

Performing a 0.2% CHX mouthwash significantly reduces the duration of PEB. Subgingival irrigation with 1% CHX didn’t increase the efficacy of the mouthwash while supragingival irrigation even decreased this efficacy, probably due to the influence of these maneuvers on the onset of bacteraemia.

**Clinical Relevance:**

These results confirm the suitability of performing a mouthwash with 0.2% CHX before tooth extractions in order to reduce the duration of PEB. This practice should perhaps be extended to all dental manipulations.

**Trial Registration:**

Clinicaltrials.gov NCT02150031

## Introduction

For several decades, the haematogenous spread of bacteria from the oral cavity has been considered a decisive factor in the pathogenesis of 10% to 15% of episodes of infective endocarditis (IE), suggesting that certain dental procedures may represent a significant risk factor. A review of the literature revealed a prevalence of positive blood cultures after dental extractions that varied between 30% and 76% in children and between 58% and 100% in adults [1]. Although notable improvements in IE diagnosis and treatment have been made, in-hospital mortality has changed little in the last four decades; this mortality rate remains close to 20% [2].

Since the AHA published its first protocol for the prevention of IE associated with dental procedures, numerous expert committees in different countries have drawn up different prophylactic regimens, many of which have subsequently been revised and modified based on subsequent epidemiological and clinical studies [3]. Recently, Dayer *et al*. [4] demonstrated that the incidence of IE had increased significantly in England since introduction of the 2008 NICE guidelines, which recommended that antibiotics should not be prescribed to prevent IE. Facing this dangerous situation, NICE announced it is to review immediately its guidance on the use of antibiotics to prevent IE [5].

In 1977, in their protocol for the prevention of IE, the American Heart Association (AHA) suggested first that disinfection of the gingival sulcus must be performed as a complement to antibiotic prophylaxis in patients considered to be at risk of IE [6]. This practice was included by the AHA and adopted by other expert committees such as the British Society for Antimicrobial Chemotherapy (BSAC) in subsequent prophylactic regimens [7,8]. In 1992, the BSAC specified the presentation and concentration of chlorhexidine (CHX) that should be used before starting the dental procedure: 1% gel at the gingival margin or 0.2% mouthwash for five minutes [7]. In 1997, the AHA recognised the need to use antiseptic mouthwashes (CHX or povidone iodine) prior to dental manipulations, although they recommended against the use of gingival irrigators and against the continuous use of antiseptics in order to avoid the selection of resistant micro-organisms [8].

In 2006, the BSAC recommended a single mouthwash with 0.2% CHX gluconate (10 ml for 1 minute) before performing dental procedures associated with bacteraemia in patients at risk of IE [9]. In contrast, in 2007, the AHA did not recommend the use of any antiseptic prophylaxis protocol [10]. In 2008, the National Institute for Health and Clinical Excellence (NICE) of the United Kingdom performed a systematic review of the antimicrobial prophylaxis protocols for IE and reported that: “CHX used as an oral rinse does not significantly reduce the level of bacteraemia following dental procedures” [11]. This conclusion was reached after analysis of numerous studies on the efficacy of prophylaxis with CHX for the prevention of post-dental manipulation bacteraemia [12–18]. However, those studies presented significant methodological differences not only in the dental procedures performed, but also in the concentration of CHX applied and the method of application of the antiseptic solution (mouthwash and/or irrigation), making comparison of the results of the different series difficult [19].

There are few studies that have analysed the efficacy of the mouthwash of 0.2% CHX (the concentration recommended by the BSAC [9]) in the prevention of post-extraction bacteraemia (PEB) [15,17,20–22], the dental procedure associated with the highest risk of bacteraemia [23]. Despite that some studies evaluated the effect of local irrigation with CHX after different dental manipulations, only two papers analysed the efficacy of CHX irrigation in preventing the PEB, one of them with 1% of CHX [13] and the other at a concentration of 0.2% [14]. Only Yamalik *et al*. [24] studied the combination of local irrigation of the tooth and mouthwash with CHX in the prevention of PEB, but with a really low concentration of CHX of only 0.02%.

The objective of this study was to investigate how different application protocols with CHX condition the prevalence, duration and aetiology of bacteraemia secondary to a simple and single tooth extraction. The hypothesis established was the following: antiseptic prophylaxis with CHX could decrease the PEB, and its method of application may have influence on its efficacy.

## Patients and Methods

This was a randomised, double blind parallel study on the efficacy of the CHX for the prevention of the PEB. The project was approved by the Ethics Committee of Clinic Investigation of Galicia (CEIC, Spain) registered with the number 2008/202. The protocol for this trial and supporting CONSORT checklist are available as supporting information; see S1 CONSORT Checklist and S1 and S2 Protocols. It was registered in Clinicaltrials.gov with the reference NCT02150031 and can be accessed at the following URL: http://clinicaltrials.gov/show/NCT02150031. Due to the big number of patients needed to accomplish the objective of the study and the technical difficulties, the register was done after the enrollment of the volunteers. Nevertheless, the authors confirm that all ongoing and related trials for this intervention are registered. All procedures done in the experiments were oral and written explained to all the patients. In addition, written informed consent was obtained.

### Selection of the study group

**Fig 1:**
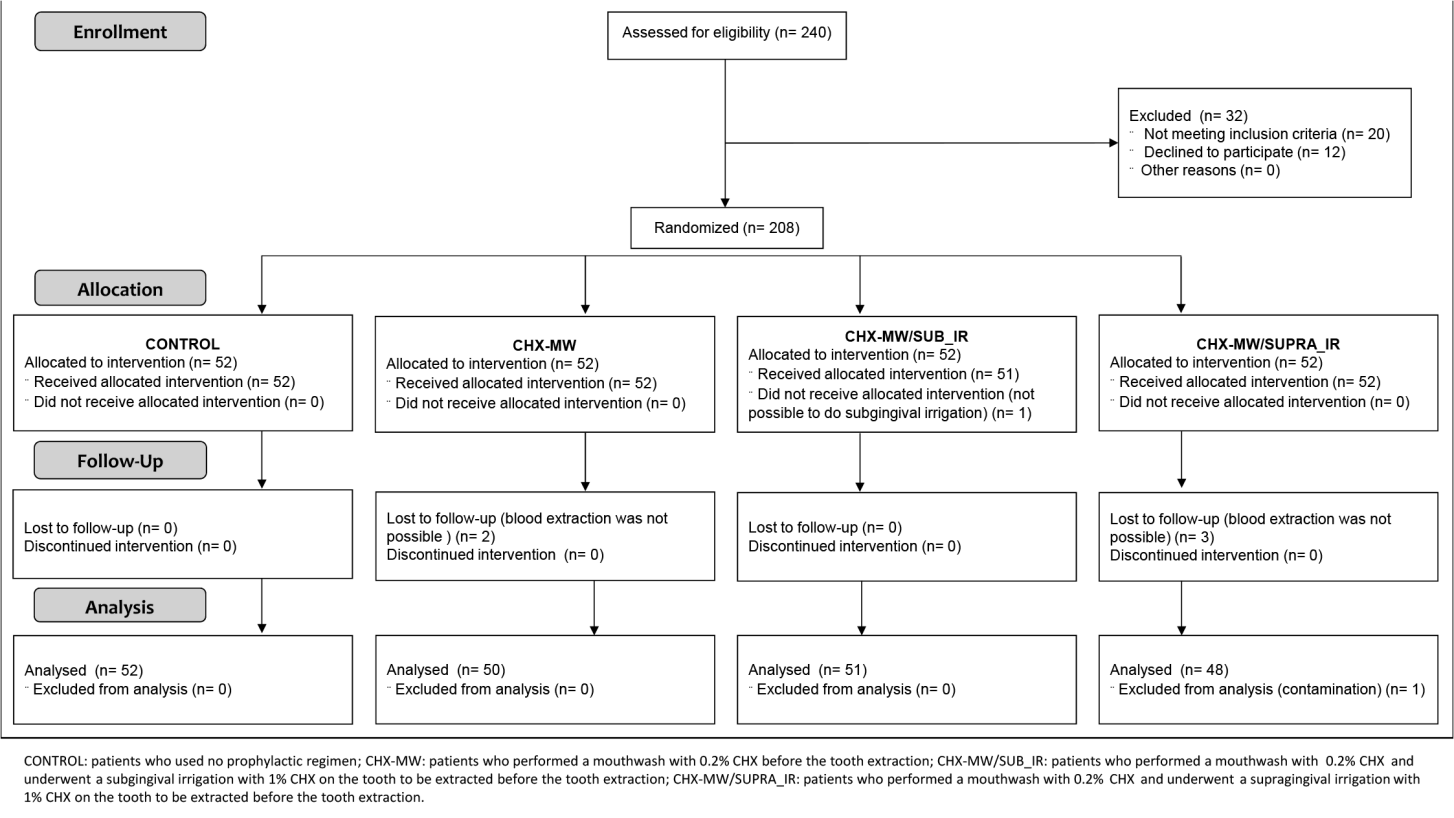
Flow diagram of the study with enrollment, allocation, follow-up and analysis of participants.

The study group was formed of 240 patients attending to the Department of Stomatology and Maxillofacial Surgery of the Santo Antonio General Hospital (Oporto, Portugal) between 2010 and 2012 needing from a simple and single tooth extraction. The dental interventions were performed under local anaesthesia by the same calibrated clinician. The following exclusion criteria were applied: patients under 18 years of age; antibiotic treatment and/or routine use of oral antiseptics in the previous three months; and any type of congenital or acquired immunodeficiency or other disease that could favour the onset of infection or haemorrhagic complications. Applying these criteria, 208 patients were selected and were randomly (using the closed envelope technique) distributed into four groups (Fig 1):

- Control group (CONTROL group): 52 patients who used no prophylactic regimen.
- CHX mouthwash group (CHX-MW group): 52 patients who performed a mouthwash with 0.2% CHX (10 ml for 1 minute) (Oraldine Perio, Johnson and Johnson, Madrid, Spain) before the tooth extraction.
- CHX mouthwash/subgingival irrigation group (CHX-MW/SUB_IR group): 52 patients who performed a mouthwash with 0.2% CHX (10 ml for 1 minute) (Oraldine Perio). After that, they underwent a subgingival irrigation with 1% CHX on (1.8 ml for 1 minute) the tooth to be extracted; the irrigation was done with the Heraeus Citoject Intraligamental Syringe (Kulzer Heraeus S.A., Madrid, Spain) at six points on each tooth (three points on the vestibular surface and three on the palatine surface).
- CHX mouthwash/supragingival irrigation group (CHX-MW/SUPRA_IR group): 52 patients who performed a mouthwash with 0.2% CHX (10 ml for 1 minute) (Oraldine Perio). After that, they underwent supragingival irrigation with 1% CHX (10 ml for 1 minute) on the tooth to be extracted; the irrigation was done continuously around the tooth to be extracted with a conventional syringe of 10 ml (BD Discardit II, Becton Dickinson S.A., Spain).

The mouthwash and subgingival or supragingival irrigation were performed immediately before injection of the local anaesthetic.

### Evaluation of the oral health status

After recording the gender and age of each patient, a single dentist performed an intraoral examination two days before the intervention, collecting the following information: plaque deposits (simplified Greene and Vermillion oral hygiene index) [25], calculus deposits (Ramfjord calculus index) [26], presence of gingival bleeding (Löe and Silness gingival index) [27], depth of gingival sulcus/periodontal pocket (Ramfjord index) [26], degree of tooth mobility (Ramfjord tooth mobility index) [26], number of caries (including root remnants), and presence of submucosal abscesses, fistulae and periapical foci detected clinically and/or radiologically. Each patient was assigned an overall oral health status using a scale previously designed and validated by the authors, which incorporates dental and periodontal health criteria [28]. The overall oral health scale has a score range between 0 ("healthy mouth") and 3 ("diseased mouth"). Furthermore, the type of tooth extraction and the reason for the extraction were also recorded for each patient.

### Characteristics of the anaesthetic technique

Local anaesthesia was administered to all patients using conventional techniques (regional block and/or infiltration). The anaesthetic employed was lidocaine plus adrenaline (1:100,000) and not more than two cartridges were used in any patient. The anaesthetic technique and the tooth extraction were done by a clinician who was not aware of the study design and objectives.

### Collection of samples for blood cultures

**Fig 2:**
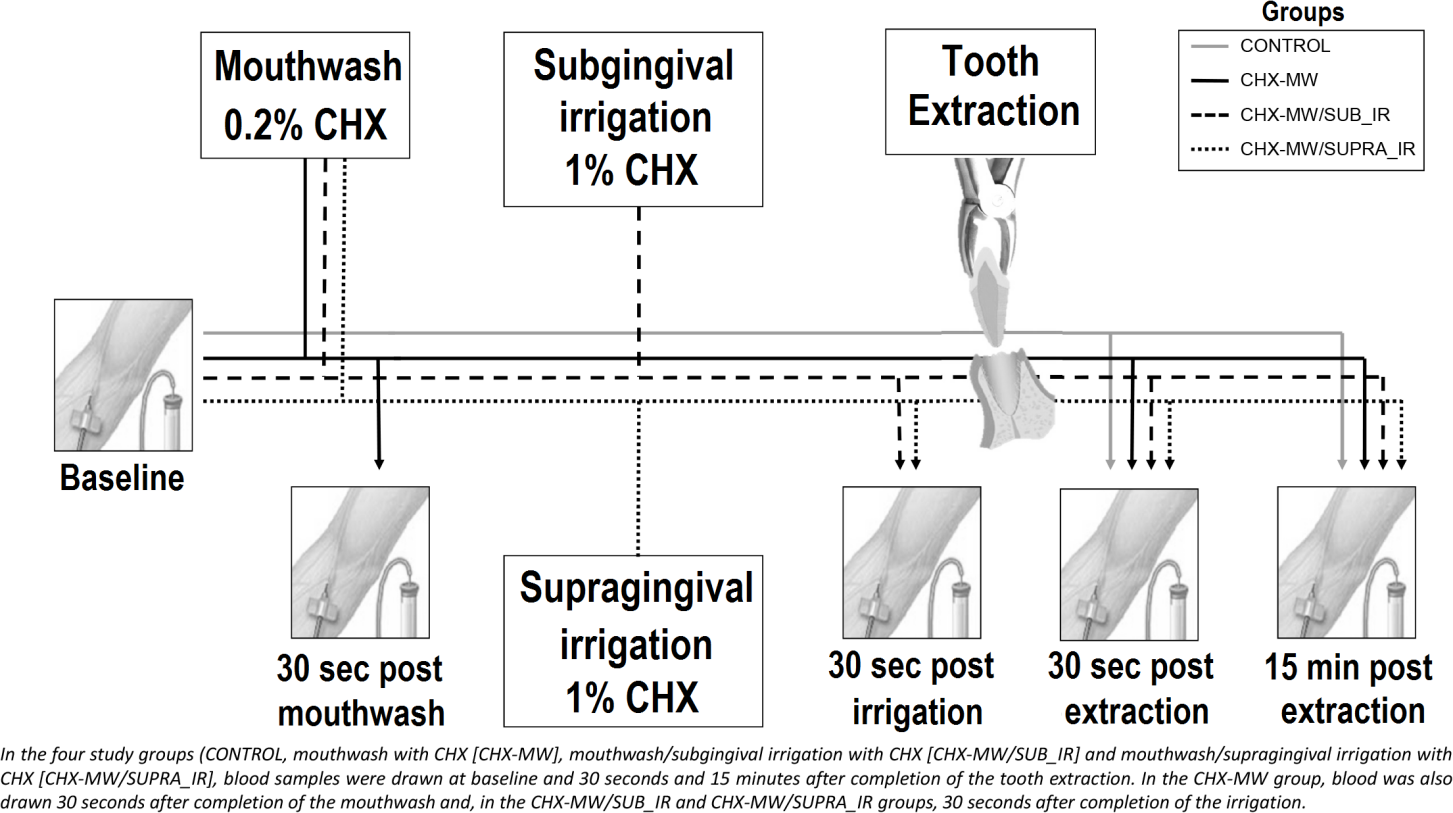
Protocol for the collection of blood samples for microbiological processing.

The prevalence of baseline bacteraemia was determined by collection of a peripheral venous blood sample (10 ml) from each patient before performing any manipulation. The prevalence of bacteraemia secondary to a mouthwash alone, a mouthwash/subgingival irrigation and a mouthwash/supragingival irrigation was determined by the collection of a peripheral blood sample (10 ml) 30 seconds after each of these actions. Further samples (10 ml) were drawn 30 seconds and 15 minutes after completion of the tooth extraction in order to determine the prevalence and duration of PEB (Fig 2).

Intravenous access was established using an 18–22 gauge "angiocath" catheter (Becton Dickinson, Sparks, MD, USA) inserted in the antecubital fossa or dorsum of the hand after disinfection of the area with alcohol and povidone iodine. The catheter was flushed with 3 ml of saline after each extraction and the first 2 ml of blood were discarded. Equal volumes of each sample were inoculated into two bottles containing aerobic and anaerobic culture media (Bactec Plus, Becton Dickinson), and the bottles were immediately transferred to the laboratory. The whole process of manipulation and transport of the samples were performed in accordance with the recommendations of the Spanish Society of Infectious Diseases and Clinical Microbiology [29].

### Microbiological analysis of the blood cultures

The blood samples were injected into the blood culture bottles, being processed in the Bactec 9240 (Becton Dickinson). Gram stain was performed on all positive cultures. The positive aerobic blood cultures were subcultured on blood agar and chocolate agar in an atmosphere with 5%-10% CO_2_, and on MacConkey agar under aerobic conditions. The same protocol was used for the positive anaerobic blood cultures but included subculture on Schaedler agar and incubation under anaerobic conditions. The bacteria isolated were identified using the battery of biochemical tests provided by the Vitek system (bioMérieux Inc., Hazelwood, Missouri, USA) for gram-positive bacteria, *Neisseria* spp./*Haemophilus* spp. and obligate anaerobic bacteria. Applying the Ruoff criteria [30], *Streptococcus viridans* were classified into five groups: *mitis*, *anginosus*, *mutans*, *salivarius* and *bovis*.

### Statistical analysis

To calculate an "a priori" sample size, the following statistical criteria were established: an effect size of 0.3, an alpha error of 0.04 and a statistical power of 95%. Assuming these criteria and the possible application of the Chi-squared test, a sample size of 50 subjects per group was required (a total of 200 subjects). We established an effect size of 0.3, which means we could detect differences greater than 18% between the prevalence of PEB in the “control group” and the “treated group”. The sample size calculation was performed using the program G*Power 3.1.5.

The results were analysed using the PASW statistical package version 21 for Windows (SPSS Inc., Chicago, USA) by an investigator who was blinded to the type of interventions analysed. Comparison of the prevalence of baseline bacteraemia with the prevalence detected after the different applications of CHX was performed using the McNemar test. Comparison of the prevalence of PEB at 30 seconds and 15 minutes after the tooth extraction between the different groups (control, CHX-MW, CHX-MW/SUB_IR and CHX-MW/SUPRA_IR) was performed using 4x2 contingency tables and the Chi-squared test. A *p* value less than 0.05 was considered statistically significant. Only in the case in which it was proved heterogeneity (association) between the different groups (this only happened in the prevalence of PEB at 15 minutes), 2x2 contingency tables were analysed using the Chi-squared test with Bonferroni correction. For pairwise comparisons, a *p* value less than 0.008 was considered statistically significant.

Basing on previous recommendations [31,32], an intention-to-treat (ITT) analysis was performed and afterwards these results were compared with those obtained in the analysis excluding missing data; in the present study, there were missing data of 7 subjects. In an ITT analysis, all randomized patients are included in the analysis in their assigned groups regardless of all considerations, including whether they in fact received the designated intervention [31,32]. In the ITT analysis, the strategy applied for dealing with missing data was the “extreme case analysis” [32], in which all missing subjects had negative blood cultures (at baseline and post-manipulation).

## Results

From the initial study group of 208, seven of them were lost, leaving a final number of 201 participants. The lost volunteers were one of them from the CHX-MW/SUB_IR, two in CHX_MW and four patients in CHX-MW/SUPRA_IR. Five of the patients were lost because of the impossibility to do a blood collection due to arteriospasm. Besides, one patient was excluded of the analysis for a possible blood sample contamination. In the group of CHX_MW/SUB_IR, an additional subject was lost due to the impossibility to do a subgingival irrigation motivated by the lack of keratinized gum surrounding the tooth (Fig 1). The clinical characteristics of the four groups were detailed in Table 1 (primary data in S1 Dataset).

**Table 1:** Clinical characteristics of the four study groups.

CLINICAL MEASURES	STUDY GROUPS				
	TOTALn (%)	CONTROLn (%)	CHX-MWn (%)	CHX-MW/SUB_IRn (%)	CHX-MW/SUPRA_IRn (%)
**Age (mean** ± **SD)**	46.7 ± 16.7	42.4 ± 17.8	48.7 ± 17.0	48.7 ± 17.1	47.4 ± 14.4
**Gender**					
Men	87 (43.3%)	21 (40.4%)	14 (28.0%)	25 (49.0%)	27 (56.2%)
Women	114 (56.7%)	31 (59.6%)	36 (72.0%)	26 (51.0%)	21 (43.8%)
**Oral Health**					
Grade 0	15 (7.5%)	9 (17.3%)	2 (4.0%)	2 (3.9%)	2 (4.2%)
Grade 1	68 (33.8%)	19 (36.6%)	12 (24.0%)	18 (35.3%)	19 (39.5%)
Grade 2	95 (47.3%)	22 (42.3%)	25 (50.0%)	23 (45.1%)	25 (52.1%)
Grade 3	23 (11.4%)	2 (3.8%)	11 (22.0%)	8 (15.7%)	2 (4.2%)
**Tooth Extracted**					
Incisor or Canine	47 (23.4%)	9 (17.3%)	13 (26.0%)	9 (17.6%)	16 (33.3%)
Premolar or Molar	154 (76.6%)	43 (82.7%)	37 (74.0%)	42 (82.4%)	32 (66.7%)
**Reason for Extraction**					
Periodontitis	37 (18.4%)	6 (11.5%)	16 (32.0%)	7 (13.7%)	8 (16.7%)
Caries or others	164 (81.6%)	46 (88.5%)	34 (68.0%)	44 (86.3%)	40 (83.3%)

*SD = standard deviation*.

The prevalence of bacteraemia at baseline was 2%. The prevalences of bacteraemia secondary to the CHX-MW, to the CHX-MW/SUB_IR and to the CHX-MW/SUPRA_IR were 4%, 12% and 6%, respectively. The differences in the percentage of positive blood cultures at baseline and immediately after subgingival irrigation showed a trend to statistical significance (*p* = 0.063).

**Fig 3:**
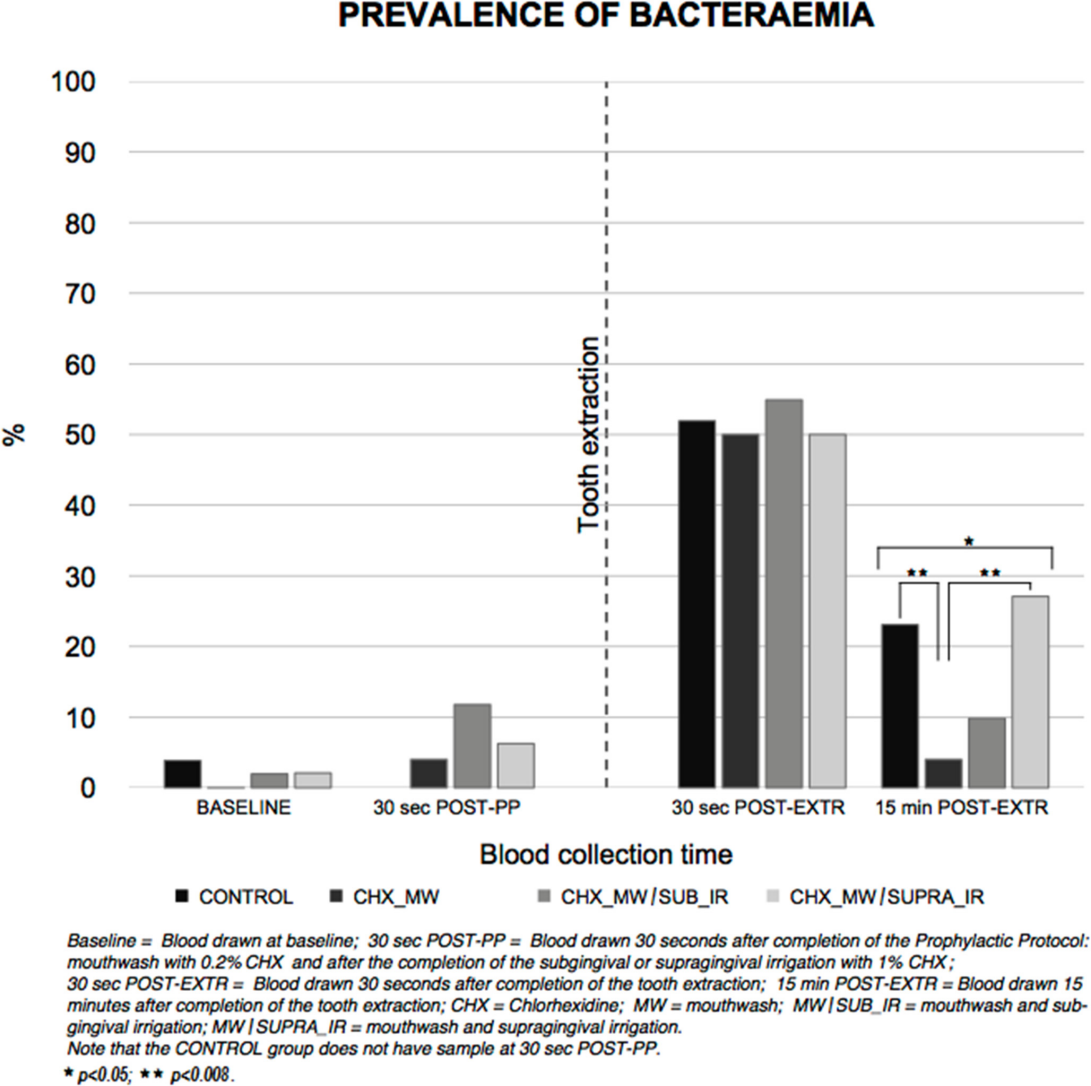
Prevalence of bacteraemia at baseline, after the mouthwash with 0.2% CHX, after a mouthwash with 0.2% CHX/subgingival irrigation with 1% CHX, after a mouthwash with 0.2% CHX/supragingival irrigation with 1% CHX and after tooth extraction in the four study groups: CONTROL group (n = 52 patients), CHX-MW group (n = 50 patients), CHX-MW/SUB_IR group (n = 51 patients) and CHX-MW/SUPRA_IR group (n = 48 patients).

The prevalences of PEB at 30 seconds after tooth extraction were similar between the different groups: 52% in the CONTROL group, 50% in the CHX-MW group, 55% in the CHX-MW/SUB_IR group and 50% in the CHX-MW/SUPRA_IR group (Fig 3, primary data in S1 Dataset); these results were independent of the score of overall oral health previously established. There were statistically significant differences in the prevalence of PEB at 15 minutes after tooth extraction between the four study groups (*p* = 0.004). In 2x2 contingency tables, this prevalence was significantly higher in the CONTROL group than in the CHX-MW group (23% *versus* 4%; *p* = 0.005). However, no differences were found between the CONTROL group and either the CHX-MW/SUB_IR group (23% *versus* 27%) or the CHX-MW/SUPRA_IR group (23% *versus* 10%). No significant differences were detected between the CHX-MW and CHX-MW/SUB_IR groups in the percentage of positive post-extraction blood cultures at 15 minutes after tooth extraction (4% *versus* 10%). On the contrary, the prevalence of PEB at 15 minutes was significantly lower in CHX-MW group in comparison to that detected in the CHX-MW/SUPRA_IR group (4% *versus* 27%; *p* = 0.002).

The results derived from the ITT analysis were similar to those obtained in the analysis excluding missing data, mainly due to the low number of cases with missing data (only 7 subjects).

Table 2 shows the principal groups of bacteria identified in the positive blood cultures in the CONTROL, CHX-MW, CHX-MW/SUB_IR and CHX-MW/SUPRA_IR groups. Streptococci (particularly the *S*. *viridans*) were the most frequently identified bacteria in the positive blood cultures, with percentages that varied between 69% (in the CHX-MW/SUPRA_IR group) and 79% (in the CHX-MW/SUB_IR group). S. *viridans* were predominantly *S*. *mitis* group in the majority of groups (43% in the CONTROL group, 46% in the CHX-MW group and 59% in the CHX-MW/SUPRA_IR group). With the exception of the CHX-MW/SUPRA_IR group, the frequency of obligate anaerobic bacteria was similar in the remaining groups, with 11% in the CONTROL group, 12% CHX-MW/SUB_IR group and 17% in the CHX-MW group.

**Table 2:** Prevalence of bacteria identified in the positive blood cultures in the CONTROL group (n = 46 isolates), CHX-MW group (n = 35 isolates), CHX-MW/SUB_IR group (n = 33 isolates) and CHX-MW/SUPRA_IR group (n = 42).

BACTERIA	CONTROL% of isolates	CHX-MW% of isolates	CHX-MW/SUB_IR% of isolates	CHX-MW/SUPRA_IR% of isolates
*Streptococcus* spp.	72	71	79	69
*Staphylococcus* spp.	6	0	0	2
*Neisseria* spp.	2	0	0	7
Obligate anaerobes	11	17	12	2
HACEK Group	0	6	0	2
Other bacteria	9	6	9	17

CHX = chlorhexidine; MW = mouthwash; SUB = subgingival; SUPRA = supragingival; IR = irrigation; HACEK Group = acronymic designation for a group of gram-negative bacteria that includes *Haemophilus* spp., *Actinobacillus actinomycetemcomitans*, *Cardiobacterium hominis*, *Eikenella corrodens* and *Kingella* spp.

Forty six isolates in the CONTROL group blood cultures

33 viridans group streptococci (20 *S*. *mitis* group, 6 *S*. *anginosus* group, 3 *S*. *mutans* group, 3 *S*. *salivarius* group and 1 *S*. *bovis* group); 3 *Staphylococcus* spp. (1 *S*. *hominis* and 2 *S*. *simulans*); 1 *Neisseria* spp. (1 *N*. *cinerea*); 5 obligate anaerobes (2 *Propionibacterium* spp. [1 *P*. *acnes*, 1 *Propionibacterium* spp.], 1 *Peptostreptococcus* spp., 1 *Fusobacterium* spp. and 1 *Bacillus* spp.*)*; and 4 other bacteria (2 *Actinomyces* spp. and 2 *Micrococcus* spp.).

Thirty five isolates in the CHX-MW group blood cultures

24 viridans group streptococci (16 *S*. *mitis* group, 4 *S*. *anginosus* group, 2 *S*. *mutans* group and 2 *S*. *salivarius* group) and 1 *Streptococcus* non-viridans group; 6 obligate anaerobes (2 *Prevotella* spp., 2 *Fusobacterium* spp., 1 *Eubacterium* spp. and 1 *Bacillus* spp.); 2 HACEK group (2 *Eikenella* spp.); and 2 other bacteria (2 *Actinomyces* spp.).

Thirty three isolates in the CHX-MW/SUB_IR group blood cultures

25 viridans group streptococci (9 *S*. *mitis* group, 10 *S*. *anginosus* group and 6 *S*. *salivarius* group) and 1 *Streptococcus* non-viridans group; 4 obligate anaerobes (1 *Peptostreptococcus* spp., 1 *Prevotella* spp., 1 *Fusobacterium* spp. and 1 *Bacillus* spp.); and 3 other bacteria (3 *Actinomyces* spp.).

Forty two isolates in the CHX-MW/SUPRA_IR group blood cultures

29 viridans group streptococci (25 *S*. *mitis* group, 2 *S*. *anginosus* group and 2 *S*. *salivarius* group); 1 *Staphylococcus* spp. (coagulase-negative); 3 *Neisseria* spp. (3 *N*. *sicca*); 1 obligate anaerobe (1 *Bacillus* spp.); 1 HACEK group (1 *Haemophilus* spp.) and 7 other bacteria (1 *Corynebacterium* spp. and 6 *Actinomyces* spp.).

## Discussion

The onset of an episode of bacteraemia constitutes an essential phase in the pathogenesis of certain focal infections of possible oral origin, such as IE [23,33]. At the present time, there is debate within the scientific community about the suitability of antibiotic prophylaxis protocols for the prevention of IE secondary to dental manipulations [34,35]. This situation is due to major controversies regarding the risk of developing IE of oral origin, the clinical repercussions of bacteraemia of oral origin, and the effectiveness of antibiotic prophylaxis in terms of risk- and cost-benefit [19,35].

The principal objective of prophylaxis with local antiseptics is to reduce the bacterial load present in the oral cavity at the time of starting the manipulation, in order to minimise the risk of developing a bacteraemia [36,37]. At the present time, further scientific evidence of the efficacy of the prophylactic CHX regimens and the investigation of new antiseptic protocols [19] is required. This is not only to establish the suitability of use of this antiseptic as a complementary measure, but also possibly to establish what could be a future alternative to antibiotic prophylaxis. To best of authors´ knowledge, any other study in the literature has determined whether the effect of a mouthwash with 0.2% CHX on the prevalence of PEB could be enhanced by the addition of irrigation with CHX, being this the first study which evaluates three different types of applications of CHX (mouthwash, mouthwash plus subgingival irrigation and mouthwash plus supragingival irrigation).

There are few studies in the literature on the efficacy of CHX mouthwash for the prevention of PEB [13–15,17,18,20–22,24,38]. Recent studies from Maharaj *et al*. [20] and Duvall *et al*. [38] didn’t find significant differences in the prevalence of PEB between doing or not a prophylactic mouthwash with 0.2% and 0.12% CHX, respectively, being in the latter even higher than placebo [38]. On the other hand, Tomás *et al*. [17] investigated the effect of “passive lavage” with 0.2% CHX for 1 minute on the prevalence of PEB in a series of physically and/or mentally disabled patients undergoing tooth extractions under general anaesthesia. Compared to the control group, the results revealed that the application of antiseptic prophylaxis produced a significant reduction in the percentage of positive post-extraction blood cultures detected at 30 seconds (96% *versus* 79%). In the present series, the 0.2% CHX mouthwash (10 ml for 1 minute) had no effect on the prevalence of PEB at 30 seconds. The discrepancies observed between the two studies could be due to methodological differences between the mouthwash techniques used (“passive” lavage with 50–60 ml *versus* “active” mouthwash with 10 ml) or to possible local changes provoked by certain anaesthetic agents used in general anaesthesia [39].

Some authors [16] considered that the inefficacy of antiseptic prophylaxis in the prevention of PEB could be because the mechanical action of rinsing might favour the passage of oral bacteria into the bloodstream. However, there is no reference to support the hypothesis [40] that the mechanical action of rinsing can cause bacteraemia. In the present series, the CHX mouthwash didn’t induce episodes of bacteraemia (only 4% of the patients presented positive post-mouthwash blood cultures compared to 2% at baseline).

In a series previously published by Tomás *et al*. [17], the effect of “passive lavage” with 0.2% CHX (0.2% for 1 minute) was investigated not only on the prevalence of PEB but also on its duration (at 15 minutes and 1 hour after completion of the intervention). Compared to the control group, there was a significant reduction in the duration of PEB after the application of antiseptic prophylaxis (at 15 minutes = 64% *versus* 30% and at 1 hour = 20% *versus* 2%) [17]. Recently, Ugwumba *et al*. [22] found that doing a mouthwash with 0.2% CHX produced a reduction in the prevalence of bacteraemia in regard with the no prophylaxis group (27.1% *versus* 52.4%) in the first 15 minutes after the tooth extraction. Coinciding with these results [22], in the present series the 0.2% CHX mouthwash significantly reduced the duration of the bacteraemic episode (the percentage of positive post-extraction blood cultures detected at 15 minutes changed from a 23% in the control group to a 4% in the mouthwash group).

It has been stated that supra- and subgingival plaque represents the principal microbial niche for the development of PEB [13,14]. A number of *in vivo* series have demonstrated the elevated antimicrobial activity of 0.2% CHX on supragingival plaque [41,42]. Although a single mouthwash with CHX doesn’t reach the apical border of the subgingival plaque in the gingival sulcus or in the periodontal pocket [43], some authors have demonstrated a bactericidal effect of a CHX mouthwash on the subgingival microbiota [44]. Thus, in agreement with results previously reported by other authors using other antiseptics [45], a mouthwash with 0.2% CHX could favour a reduction in the size of the bacterial inoculum that enters the bloodstream during tooth extraction, facilitating its rapid elimination by the immune system. This would explain the results obtained in the present study on the effect of a mouthwash with 0.2% CHX on the duration of PEB.

There are few studies in the literature that have investigated the efficacy of subgingival irrigation with CHX for the prevention of PEB [13,14,24]. MacFarlane *et al*. [13] and Rahn *et al*. [14] detected a significantly lower prevalence of bacteraemia associated with certain dental manipulations in those patients on whom irrigation of the gingival sulcus was performed with different antiseptics (CHX or povidone iodine). These authors [13,14] suggested that the reduction in the prevalence of post-dental manipulation bacteraemia was due to the bactericidal action of the antiseptics rather than the mechanical effect of the irrigation. In the design of these studies, the patients held the antiseptic solution in the oral cavity for 2 minutes after subgingival irrigation [13,14,24], whereas the patients in the present series first performed the mouthwash with 0.2% CHX (10 ml for 1 minute) and then underwent subgingival irrigation with 1% CHX. The results of the present series showed that the combination of the mouthwash with subgingival irrigation didn’t reduce the prevalence of PEB (at 30 seconds) neither its duration (at 15 minutes) compared to mouthwash alone or no antiseptic prophylaxis.

The degree of penetration of CHX into the gingival sulcus and its antibacterial effect could be determined by various factors, such as the presence of subgingival calculus, the type of irrigation tip used and even the way the tip is positioned over the gingival sulcus [46]. An important methodological difference between the present series and previous studies that may have affected the results obtained was detected. In the studies by McFarlane *et al*. [13] and Yamalik *et al*. [24] the irrigation was performed with a plastic syringe and a blunt-tipped needle, irrigating with a total volume of 10 ml of antiseptic. In the present series, the subgingival irrigation was performed with an intraligamental anaesthesia syringe, which permits to control the force of application and the volume of CHX applied at each point (six points per tooth), and the total volume of CHX used, which was of approximately 2 ml.

It has been stated that the most effective method of application of an antiseptic to reduce the prevalence of post-dental manipulation bacteraemia is irrigation of the gingival sulcus prior to the dental treatment [14,47]. However, in 1997, the AHA recommended against the application of antiseptics by means of gingival irrigators [8], probably assuming that subgingival irrigation could favour the passage of oral bacteria into the bloodstream. However, few studies have been published on this subject and their results are contradictory. Witzenberger *et al*. in 1982 [48] and Lofthus *et al*. in 1991 [49], investigated the prevalence of bacteraemia secondary to subgingival irrigation in patients with periodontal pockets ≥4 mm and macroscopic bleeding. While Witzenberger *et al*. [48] detected no positive post-irrigation blood cultures with povidone iodine, Lofthus *et al*. [49] reported bacteraemia in 30% of cases (6 of 20 patients) at 2 minutes after completing the irrigation, and found no significant differences between the use of CHX or sterile water as the irrigation solution; it should also be noted that, prior to the irrigation, Lofthus *et al*. detected a 10% prevalence of baseline bacteraemia (2 of 20 patients). In the present series, the subgingival irrigation also produced an important increase in the prevalence of bacteraemia compared to the baseline from 2% to 12%.

Any study about the prevalence of PEB after supragingival irrigation has been found. In the present series, a mouthwash with 0.2% CHX plus supragingival irrigation with a continuous flow of 1% CHX, all around the tooth to be extracted, didn’t reduce the prevalence of PEB (at 30 seconds) neither its duration (at 15 minutes). Conversely, the CHX-MW was more effective at reducing bacteraemia than the CHX-MW/SUPRA_IR protocol 15 minutes after the tooth extraction. These differences probably were due to the supragingival irrigation flow disturbed the supragingival plaque attached to the tooth. These free bacteria may be more susceptible to come into the alveolus after dental extraction than when they are embedded in the structured dental plaque biofilm.

In numerous studies on bacteraemia secondary to tooth extractions, *Streptococcus viridans* were the bacteria most frequently isolated in the post-extraction blood cultures, accounting from 60% to 75% of all bacteria identified [15,33,50]. In agreement with those results, approximately 69%-79% of the isolates were of *Streptococcus* spp. in the present study, with a predominance of viridans group streptococci *(S*. *mitis* group). According to Parahitiyawa *et al*. [23], the absence of bacterial diversity in bacteraemia of oral origin is probably due to the presence of virulence factors that favour the entry of certain bacteria into the bloodstream and rapid bacterial clearance by host defence mechanisms, as well as the low level of detection of certain pathogens by the conventional methods typically used in clinical laboratories. Nowadays, some authors have shown the limitations of the culture technique to identify the bacterial diversity of PEB [51]. Recently, Benítez-Paez *et al*. [51] using pyrosequencing of 16S rRNA genes, detected an extraordinarily high bacterial diversity of PEB in contrast with conventional culture-dependent methods. However, this molecular technique underestimated the prevalence and duration of PEB, probably because the low bacterial load present in blood samples, thus limiting the recovery of the DNA required for PCR amplification.

In conclusion, a 0.2% CHX mouthwash (10 ml for 1 minute) significantly reduced the duration (at 15 minutes) of bacteraemia secondary to tooth extraction under local anaesthesia. Subgingival irrigation with 1% CHX didn’t increase the efficacy of the mouthwash while supragingival irrigation even decreased this efficacy, probably due to the influence of these maneuvers on the development of bacteraemia. These results confirm the suitability of performing a mouthwash with 0.2% CHX before tooth extractions in order to reduce the duration of PEB. This practice should perhaps be extended to all dental manipulations.

## Supporting Information

S1 CONSORT Checklist CONSORT Checklist of the present study. (DOCX)

S1 Dataset Primary data table. (XLSX)

S1 Protocol Trial Protocol of the study. Post-tooth extraction bacteraemia: a randomized clinical trial on the efficacy of chlorhexidine prophylaxis.
(DOCX)

S2 Protocol Trial Protocol of the study (original version in Spanish). Post-tooth extraction bacteraemia: a randomized clinical trial on the efficacy of chlorhexidine prophylaxis.
(DOCX)
