# Supplementary material for: Post-Tooth Extraction Bacteraemia: A Randomized Clinical Trial on the Efficacy of Chlorhexidine Prophylaxis
Source: PLoS One. 2015 May 8;10(5):e0124249. doi: 10.1371/journal.pone.0124249 (PMC4425363; doi:10.1371/journal.pone.0124249)
Supplement: S1 Protocol — Post-tooth extraction bacteraemia: a randomized clinical trial on the efficacy of chlorhexidine prophylaxis. (DOCX) [file pone.0124249.s003.docx]

**POST-EXTRACTION BACTERAEMIA: A RANDOMIZED CLINICAL TRIAL ON THE EFFICACY OF CHLORHEXIDINE PROPHYLAXIS**

(DIRECTOR: INMACULADA TOMÁS CARMONA)

1. OBJECTIVES

The objective of this study will be to investigate how different application protocols with chlorhexidine (CHX) condition the prevalence, duration and aetiology of bacteraemia secondary to a simple and single tooth extraction. The hypothesis established will be the following: antiseptic prophylaxis with CHX could decrease the post-extraction bacteraemia (PEB), and its method of application may have influence on its efficacy.

1. **MATERIAL, METHODS AND WORK PLAN**

This is a randomized, double blind parallel study on the efficacy of the chlorhexidine for the prevention of post-extraction bacteraemia.

- Sample size and study group selection

The study group will be formed of 200 systemically healthy adult volunteers.

- Inclusion Criteria:
- Age: over 18 years old.
- Needing a simple single tooth extraction under local anesthesia.
- Exclusion Criteria:
  - Age: lower 18 years old.
  - Routine use of antibiotic and/or oral antiseptic during the previous three months.
  - Presence of any systemic disease that could favour the onset of infection or haemorrhagic complications.

Applying these criteria, the 200 patients will be selected and will be randomly distributed into the next four groups:

- Control group (CONTROL group): 50 patients who will use no prophylactic regimen.
- CHX mouthwash group (CHX-MW group): 50 patients who will perform a mouthwash with 0.2% CHX (10 ml for 1 minute) (Oraldine Perio, Johnson and Johnson, Madrid, Spain) before the tooth extraction.
- CHX mouthwash/subgingival irrigation group (CHX-MW/SUB_IR group): 50 patients who will perform a mouthwash with 0.2% CHX (10 ml for 1 minute) (Oraldine Perio). After that, they will be submitted to a subgingival irrigation with 1% CHX (1.8 ml for 1 minute) on the tooth to be extracted; the irrigation will be performed with the Heraeus Citojet Intraligamental Syringe (Kulzer Heraeus S.A., Madrid, Spain) at six points on each tooth (three points on the vestibular surface and three on the palatine surface).
- CHX mouthwash/supragingival irrigation group (CHX-MW/SUPRA_IR group): 50 patients who will perform a mouthwash with 0.2% CHX (10 ml for 1 minute) (Oraldine Perio). After that, they will be submitted to a supragingival irrigation with 1% CHX (10 ml for 1 minute) on the tooth to be extracted; the irrigation will be performed with a continuously around the tooth to be extracted with a conventional syringe of 10 ml (BD Discardit II, Becton Dickinson S.A., Spain).
- Evaluation of the oral health status

After recording the gender and age of each patient, the same dentist will perform an intraoral examination two days before the intervention, collecting the following information: plaque deposits (simplified Greene and Vermillion oral hygiene index) [1], calculus deposits (Ramfjord calculus index) [2], presence of gingival bleeding (Löe and Silness gingival index) [3], depth of periodontal pockets (Ramfjord index) [2], degree of tooth mobility (Ramfjord tooth mobility index) [2], number of caries (including root remnants), and presence of submucosal abscesses, fistulae and periapical foci detected clinically and/or radiologically. Each patient will be assigned an overall oral health status using a scale designed by the authors and published previously; the scale incorporates dental and periodontal health criteria [4]. The overall oral health scale has a score range between 0 ("healthy mouth") and 3 ("diseased mouth"). Furthermore, the type of tooth extraction and the reason for the extraction will be also recorded for each patient.

- Characteristics of the anaesthetic technique

Local anaesthesia will be administered to all patients using conventional techniques (regional block and/or infiltration). The anaesthetic employed will be lidocaine plus adrenaline (1:100,000) and not more than two cartridges will be used in any patient. The anaesthetic technique and the tooth extraction will be done by the same clinician who will not be aware of the study design and objectives.

- Collection of samples for blood cultures

The prevalence of baseline bacteraemia will be determined by collection of a peripheral venous blood sample (10 ml) from each patient before performing any manipulation. The prevalence of bacteraemia secondary to a mouthwash and subgingival or supragingival irrigation will be determined by the collection of a peripheral blood sample (10 ml) 30 seconds after each of these actions. Further samples (10 ml) will be drawn 30 seconds and 15 minutes after completion of the tooth extraction in order to determine the prevalence and duration of post-extraction bacteraemia.

Intravenous access will be established using an 18-22 gauge "angiocath" catheter (Becton Dickinson, Sparks, MD, USA) will insert in the antecubital fossa or dorsum of the hand after disinfection of the area with alcohol and povidone iodine. The catheter will be flushed with 3 ml of saline after each extraction and the first 2 ml of blood will be discarded. Equal volumes of each sample will be inoculated into two bottles containing aerobic and anaerobic culture media (Bactec Plus, Becton Dickinson), and the bottles will be immediately transferred to the laboratory. The whole process of manipulation and transport of the samples will be performed in accordance with the recommendations of the Spanish Society of Infectious Diseases and Clinical Microbiology [5].

- Microbiological analysis of the blood cultures

The blood samples will be injected into the blood culture bottles, being processed in the Bactec 9240 (Becton Dickinson). Gram stain will be performed on all positive cultures. The positive aerobic blood cultures will be subcultured on blood agar and chocolate agar in an atmosphere with 5%-10% CO_2_, and on MacConkey agar under aerobic conditions. The same protocol will be used for the positive anaerobic blood cultures but will be included subculture on Schaedler agar and incubation under anaerobic conditions. The bacteria will be isolated and identified using the battery of biochemical tests provided by the Vitek system (bioMérieux Inc., Hazelwood, Missouri, USA) for gram-positive bacteria, *Neisseria* spp./ *Haemophilus* spp. and obligate anaerobic bacteria. Applying the Ruoff criteria [6], *Streptococcus viridans* will be classified into five groups: *mutans*, *salivarius*, *bovis*, *anginosus* and *mitis*.

- Statistical analysis

To calculate an "a priori" sample size, the following statistical criteria will be established: an effect size of 0.3, an alpha error of 0.04 and a statistical power of 95%. Assuming these criteria and the possible application of the Chi-squared test, a sample size of 50 subjects per group will be required (a total of 200 subjects). The sample size calculation will be performed using the program G*Power 3.1.5.

The results will be analysed using the PASW statistical package version 21 for Windows (SPSS Inc., Chicago, USA) by an investigator who will be blinded to the type of interventions analysed. Comparison of the prevalence of baseline bacteraemia with the prevalences detected after the different applications of CHX will be performed using the McNemar test. Comparison of the prevalence of PEB at 30 seconds and 15 minutes after the tooth extraction between the different groups (control, CHX-MW, CHX-MW/SUB_IR and CHX-MW/SUPRA_IR) will be performed using the Chi-squared test; this test also will be applied for pairwise comparisons. A *p* value less than 0.05 will be considered statistically significant. The probability after multiple comparisons will be evaluated using Bonferroni correction, applying a significant level of *p*< 0.008.

Basing on previous recommendations [7,8], an intention-to-treat (ITT) analysis will be performed after to compare the obtained results with those will describe in the analysis excluding missing data. In an ITT analysis, all randomized patients are included in the analysis in their assigned groups regardless of all considerations, including whether they in fact received the designated intervention [7,8].

1. **BIBLIOGRAPHY**

1. Greene JC, Vermillion JR. The Simplified Oral Hygiene Index. J Am Dent Assoc. 1964;68: 7-13.

2. Ramfjord SP. The Periodontal Disease Index (PDI). J Periodontol. 1967;38(Suppl): 602-610.

3. Loe H, Silness J. Periodontal Disease in Pregnancy. I. Prevalence and Severity. Acta Odontol Scand. 1963;21: 533-551.

4. Relvas M, Diz P, Seoane J, Tomás I. Oral Health Scales: design of an oral health scale of infectious potential. Med Oral Patol Oral Cir Bucal 2013;18: e664-670.

5. Loza E, Planes A, Rodríguez M. Hemocultivos. In: Cercenado E, Cantón R, editors. Procedimientos en Microbiología Clínica. Spain: Sociedad Española de Enfermedades Infecciosas y Microbiología Clínica; 2003. pp. 1-23.

6. Ruoff KL. Miscellaneous catalase-negative, gram-positive cocci: emerging opportunists. J Clin Microbiol. 2002;40: 1129-1133.

7. Altman DG. Missing outcomes in randomized trials: addressing the dilemma. Open Med. 2009;3: e51-53.

8. Sainani KL. Making sense of intention-to-treat. PM R. 2010;2: 209-213.
