# Supplementary material for: Post-Tooth Extraction Bacteraemia: A Randomized Clinical Trial on the Efficacy of Chlorhexidine Prophylaxis
Source: PLoS One. 2015 May 8;10(5):e0124249. doi: 10.1371/journal.pone.0124249 (PMC4425363; doi:10.1371/journal.pone.0124249)
Supplement: S2 Protocol — Post-tooth extraction bacteraemia: a randomized clinical trial on the efficacy of chlorhexidine prophylaxis. (DOCX) [file pone.0124249.s004.docx]

**PROTOCOLO DE INVESTIGACIÓN**

**BACTERIEMIA POST-EXODONCIA: UN ENSAYO CLÍNICO RANDOMIZADO DE LA EFICACIA DE LOS PROTOCOLOS DE PROFILAXIS CON CLORHEXIDINA**

(DIRECTORA: INMACULADA TOMÁS CARMONA)

1. OBJETIVOS

El objetivo de este estudio será investigar la prevalencia, duración y etiología de las bacteriemias secundarias a una extracción sencilla de un diente previa aplicación de tres protocolos diferentes de profilaxis con clorhexidina (CHX). La hipótesis planteada será la siguiente: la profilaxis antiséptica con CHX puede disminuir la bacteriemia post-exodoncia, estando esta eficacia condicionada por el método de aplicación.

1. MATERIAL Y MÉTODOS

Se plantea realizar un estudio aleatorizado a doble ciego de la eficacia de la CHX para la prevención de bacteriemias secundarias a la práctica de exodoncias.

- **Tamaño muestral y selección del grupo de estudio**

Asumiendo los siguientes criterios (efecto del tamaño de 0,30, error tipo alpha de 0,04 y con un poder estadístico del 95%) y la aplicación del test estadístico Chi-cuadrado, el tamaño muestral preciso será de 50 sujetos por grupo. El cálculo del tamaño muestral se realizó con el programa G*Power 3.1.5. El grupo de estudio estará formado por 200 voluntarios adultos.

-Criterios de Inclusión:

- Edad: Mayores de 18 años.
- Que precisen la exodoncia simple de un diente y que pueda efectuarse bajo anestesia local.

-Criterios de Exclusión:

- - Edad: Menores de 18 años.
  - Que hayan tomado de forma rutinaria antibióticos y antisépticos los tres meses previos al estudio.
  - Presencia de enfermedades sistémicas que puedan favorecer infecciones o complicaciones hemorrágicas.

Siguiendo los criterios anteriormente citados los 200 pacientes serán seleccionados y distribuidos aleatoriamente en cuatro grupos:

**Grupo control** (“CONTROL group”): lo formarán 50 pacientes que no usarán ningún régimen profiláctico.

**Grupo enjuague con CHX** (“CHX-MW group”): lo formarán 50 pacientes los cuales realizarán un enjuague con CHX al 0.2% (10 ml durante 1 minuto) (Oraldine Perio, Johnson and Johnson, Madrid, España) inmediatamente antes de la exodoncia.

**Grupo enjuague más irrigación subgingival** **con CHX** (“CHX-MW/SUB_IR group”): lo formarán 50 pacientes que realizarán un enjuague con CHX al 0.2% y posteriormente se les practicarán una irrigación subgingival con CHX al 1% en el diente que se extraerá; para la irrigación se utilizará la jeringa intraligamentosa Citojet de Heraeus (Kulzer Heraeus S.A., Madrid, España) en seis puntos por cada diente (tres puntos en palatino y otros tres en vestibular).

**Grupo enjuague más irrigación supragingival** **con CHX** (“CHX-MW/SUPRA_IR group”): lo formarán 50 pacientes que realizarán un enjuague con CHX al 0.2% y posteriormente se les practicarán una irrigación supragingival con CHX al 1% con una jeringa convencional de forma continua alrededor del diente que se extraerá.

- **Evaluación del estado de salud oral**

Después de recoger los datos de género y edad, dos días antes de la intervención el mismo dentista realizará un examen intraoral, recogiendo datos sobre: depósitos de placa [1], depósitos de cálculo [2], presencia de sangrado gingival [3], profundidad de surco gingival/bolsa periodontal [2], grado de movilidad dentaria [2], número de caries (incluyendo raíces remanentes) y presencia de abscesos submucosos, fistulas y focos apicales clínicos o radiológicos. A cada paciente se le asignará una puntuación sobre su estado de salud oral, utilizando una escala diseñada por los autores y publicada previamente que incorpora parámetros de salud dental y periodontal [4]. Los grados de la escala de salud oral oscilan entre grado 0 (“boca sana”) y grado 3 (“boca enferma”). Además se recogerán datos sobre el tipo de diente extraído y el motivo de la exodoncia.

- **Técnica anestésica**

En todos los pacientes se aplicarán técnicas convencionales de anestesia local (bloqueo troncular y/o infiltrativa periapical). Se utilizará lidocaína más adrenalina (en concentración 1:100,000) y no más de dos cartuchos por paciente. La técnica de anestesia y la extracción del diente la realizará siempre el mismo clínico el cual no conocerá el diseño ni objetivos del estudio.

- **Toma de las muestras sanguíneas**

En cada paciente, se tomarán varias muestras periféricas de sangre venosa (10 ml): en condiciones basales (antes de ninguna manipulación), a los 30 segundos después del enjuague y las irrigaciones subgingival o supragingival, a los 30 segundos y 15 minutos de la exodoncia.

Para el acceso intravenoso se usará un catéter de 18-22 (Becton Dickinson, Sparks, MD, USA) que se colocará en la fosa ante-cubital o en el dorso de la mano, previa desinfección del área con alcohol y povidona iodada. El catéter se lavará con 3 ml de solución salina antes de la toma sanguínea y los primeros 2 ml de sangre se desecharán. Volúmenes iguales de muestra sanguínea se inocularán en dos botes que contendrán medio de cultivo aerobio y anaerobio (Bactec Plus, Becton Dickinson), y los botes se transferirán inmediatamente al laboratorio. El procesado, la manipulación y el transporte de las muestras sanguíneas seguirán las recomendaciones de la Sociedad Española de Enfermedades Infecciosas y Microbiología Clínica [5].

- **Análisis microbiológico de los cultivos**

Las muestras sanguíneas inyectadas en los botes de cultivo se procesarán en el Bactec 9240 (Becton Dickinson). En todos los hemocultivos positivos se realizará una tinción de Gram. Los hemocultivos aeróbicos positivos se cultivarán en Agar Sangre y Agar Chocolate en una atmósfera con 5%-10% de CO_2_, y en Agar MacConkey bajo condiciones aeróbicas. El mismo protocolo se seguirá con los cultivos anaeróbicos positivos más el cultivo en Agar Schaedler e incubación en condiciones anaeróbicas. Las bacterias aisladas se identificarán usando la batería de test bioquímicos del sistema Vitek (bioMérieux Inc, Hazelwood, Missouri, USA) para bacterias Gram-positivas, *Neisseria* ssp./*Haemophilus* ssp. y bacterias anaerobias estrictas. Se aplicarán los criterios de Ruoff [6] para la identificación de los *Streptococcus viridans* en cinco grupos: *mutans, salivarius, bovis, anginosus* y *mitis*.

- **Análisis estadístico**

Los resultados se analizarán utilizando el programa estadístico PASW versión 21 para Windows (SPSS Inc., Chicago, USA) por un investigador ciego al tipo de estudio e intervención realizada. La comparación de las prevalencias de bacteriemia en condiciones basales y las detectadas después de las diferentes aplicaciones de CHX se efectuará aplicando el test de McNemar. La comparación de las prevalencias de bacteriemia a los 30 segundos y 15 minutos post-exodoncia entre los diferentes grupos de estudio (control, CHX-MW, CHX-MW/SUB_IR y CHX-MW/SUPRA_IR) se efectuará aplicando el test Chi-cuadrado. Para considerar las diferencias estadísticamente significativas, el valor *p* establecido será menor de 0,05. En las comparaciones múltiples se aplicará la corrección de Bonferroni y un valor *p*< 0,008.

En base a recomendaciones previas [7,8], se efectuará un “intention-to-treat (ITT) analysis” con la finalidad de contrastar los resultados obtenidos con aquellos descritos excluyendo los datos perdidos. En este tipo de análisis, se incluirán todos los pacientes en sus respectivos grupos independientemente de no haber recibido finalmente la intervención asignada [7,8].

1. BIBLIOGRAFÍA

1. Greene JC, Vermillion JR. The Simplified Oral Hygiene Index. J Am Dent Assoc. 1964;68: 7-13.

2. Ramfjord SP. The Periodontal Disease Index (PDI). J Periodontol. 1967;38(Suppl): 602-610.

3. Loe H, Silness J. Periodontal Disease in Pregnancy. I. Prevalence and Severity. Acta Odontol Scand. 1963;21: 533-551.

4. Relvas M, Diz P, Seoane J, Tomás I. Oral Health Scales: design of an oral health scale of infectious potential. Med Oral Patol Oral Cir Bucal 2013;18: e664-670.

5. Loza E, Planes A, Rodríguez M. Hemocultivos. In: Cercenado E, Cantón R, editors. Procedimientos en Microbiología Clínica. Spain: Sociedad Española de Enfermedades Infecciosas y Microbiología Clínica; 2003. pp. 1-23.

6. Ruoff KL. Miscellaneous catalase-negative, gram-positive cocci: emerging opportunists. J Clin Microbiol. 2002;40: 1129-1133.

7. Altman DG. Missing outcomes in randomized trials: addressing the dilemma. Open Med. 2009;3: e51-53.

8. Sainani KL. Making sense of intention-to-treat. PM R. 2010;2: 209-213.
